# Biotin

**DOI:** 10.1016/j.advnut.2024.100251

**Published:** 2024-05-31

**Authors:** Cydne A Perry, Tammy A Butterick

**Affiliations:** 1 Department of Applied Health Science, Indiana University School of Public Health, Bloomington, IN, United States; 2 Department of Veterans Affairs, Research Service, Minneapolis VA Health Care System, MN, United States; 3 Department of Neuroscinece, University of Minnesota, MN, United States; 4 Department of Food Science and Nutrition, University of Minnesota, St. Paul, MN, United States

## Introduction

This article is an updated version of a previous publication about biotin [1]. Biotin is a sulfur-containing water-soluble B-vitamin critical for human metabolism. Biotin functions as a required cofactor for enzymes involved in carboxylation reactions essential for gluconeogenesis, lipid metabolism, and amino acid catabolism. The enzymes that require biotin are acetyl CoA carboxylase (ACC) 1 and 2 (ACC2), methylcrotonyl CoA carboxylase (MCC), propionyl CoA carboxylase (PCC), and pyruvate carboxylase. Each enzyme consists of 3 conserved functional domains: biotin carboxyl carrier protein (BCCP), biotin carboxylase, and carboxyltransferase, which orchestrate a multistep process to facilitate carboxylation reactions [1]. First, biotin binds to the BCCP domain of each enzyme. This binding is catalyzed by holocarboxylase synthetase (HLCS), which attaches biotin to a lysine residue, forming biocytin. Subsequently, the biotin carboxylase domain catalyzes the transfer of carbon dioxide from bicarbonate to biocytin affixed to BCCP. The final step involves the carboxyltransferase domain, which transfers the carboxyl group from biotin to various acceptor molecules. Upon binding of cabon dioxide to these acceptor molecules, the newly formed compounds participate in critical metabolic pathways relevant to human health, such as gluconeogenesis, facilitated by mitochondrial pyruvate carboxylase; amino acid catabolism, aided by mitochondrial MCC and PCC; and lipid metabolism, influenced by cytosolic ACC1 and mitochondrial ACC2. Specifically, ACC1 is pivotal in promoting fatty acid synthesis, whereas ACC2 plays a regulatory role by inhibiting fatty acid oxidation, illustrating their complementary functions in lipid metabolism.

Early evidence suggested that biotin covalently bound to lysine on histones influenced the epigenetic regulation of gene function and chromatin structure. However, subsequent research indicated that histone biotinylation in humans is minimal, casting doubt on its significant role in modifying histone structures to regulate gene expression. More recent studies, however, propose an alternate mechanism for the role of biotin in gene expression (see Recent Research section).

## Deficiencies

Biotin deficiency due to inadequate dietary intake is rare, and severe biotin deficiency in healthy adults consuming a well-balanced diet has yet to be reported [2]. Marginal biotin deficiency, however, is common during pregnancy, and biotin catabolism during lactation has been reported [3]. Diets high in raw egg whites and individuals on total parenteral nutrition without supplemental biotin may lead to symptomatic biotin deficiency. Chronic alcohol use, smoking, anticonvulsant medications, and inflammatory bowel disease may inhibit biotin absorption and increase biotin catabolism resulting in functional biotin deficiency. Individuals with genetic deficiencies in biotinidase and HLCS experience severe biotin deficiency that lead to clinical symptoms such as hallucinations, depression, anorexia, dermatitis, impaired cellular and humoral immune function, hair loss, seizures, developmental delays, metabolic ketoacidosis, hearing loss, impaired vision, lethargy, muscle weakness, vomiting, and altered fatty acid composition.

Traditionally, biotin status has been evaluated using blood and urine samples. However, low plasma biotin may not be a reliable indicator of biotin intake or status. Decreased urinary excretion of biotin along with increased urinary excretion of 3-hydroxyisovaleric acid (3-HIA) may serve as early and sensitive indicators of biotin status. Furthermore, urinary 3-HIA excretion may be an indicator of marginal biotin deficiency and a functional marker of biotin status. Increased excretion of 3-HIA reflects the reduced activity of MCC, a biotin-dependent enzyme responsible for leucine degradation. The diminished activity of MCC leads to the accumulation of 3-methylcrotonyl-CoA, which is then converted to 3-HIA via an alternate pathway. To date, the best indicator of biotin status is the reduced activity of PCC in blood lymphocytes. However, this method is highly challenging and requires special care, handling, and storage of blood samples [4].

## Diet Recommendations

The dietary requirement for biotin is unknown. The Food and Nutrition Board (FNB) at the National Academies of Sciences, Engineering, and Medicine has found insufficient data to derive an estimated average requirement and recommended dietary allowance for biotin, thus recommendations are established as adequate intakes (AI). The FNB derived the AIs by extrapolating data from infants exclusively fed human milk and used body weight to determine the AIs for other groups. The AI for biotin is 5 μg/d for infants aged 0–6 mo, 6 μg/d for infants 7–12 mo, 8 μg/d for toddlers 1–3 y, 12 μg/d for children 4–8 y, 20 μg/d for children 9–13 y, and 25 μg/d for adolescents 14–18 y. The AI for adults aged 19 y and older and pregnant persons is 30 μg/d, and that for lactating persons is 35 μg/d [5].

## Food Sources

Biotin is widely distributed in a variety of foods, although its concentrations vary broadly. Foods high in biotin are within the protein group such as meats, fish, and eggs. In contrast, vegetables, fruits, dairy products, and grains contain considerably lower amounts of biotin or none at all [2]. The majority of biotin in food is protein bound, which may explain the abundance of biotin in animal-based food items. Biotin from food must be hydrolytically released by biotinidase before absorption, and dietary biotin is considered to be 100% bioavailable. The biotin content in food has historically been measured using nonspecific or microbial growth assays, which are not chemically specific for biotin, thus food composition tables for biotin are incomplete and the accuracy of dietary biotin intake in human populations require further substantiation.

## Clinical Uses

Pharmacologic doses of supplemental biotin are given to individuals diagnosed with HLCS and biotinidase genetic mutations. Although recent evidence suggests a potential for biotin in patients with multiple sclerosis (see Recent Research section), more research is required in the clinical use of biotin beyond genetic defects.

## Toxicity

Adverse effects or toxicity resulting from high intakes of biotin from food or supplements have not been reported in humans. Thus, the FNB is unable to establish a tolerable upper intake level for biotin [5].

## Recent Research

Recent evidence suggests that the impact of biotin on gene expression may be mediated through a multiprotein complex involving histone methylation, DNA methylation, and histone deacetylation [6]. The mechanism posits that HLCS, localized on chromatin structures, catalyzes the biotinylation of histones at specific binding sites, potentially aiding in chromatin condensation. Interestingly, a novel role for biotin has emerged in the defense against reactive oxygen species through the biotinylation of heat shock proteins in human embryonic kidney cells [7].

High-dose supplemental biotin has been reported to improve clinical outcomes in patients with progressive multiple sclerosis [8]. However, a randomized, double-blind, placebo-controlled, phase 3 clinical trial reported that high-dose biotin did not significantly improve disability or walking in patients with progressive multiple sclerosis [9]. Thus, more research is required in the clinical use of biotin for this degenerative disease.

A controlled-feeding study performed in pregnant, lactating, and nonpregnant/nonlactating individuals used liquid chromatography mass spectrometry methodology to quantify the biotin content in the meals provided throughout the 12-wk intervention period [3]. Outcomes revealed that dietary biotin ranged from 13 to 101 μg/d, providing 57 μg of dietary biotin/d. Meals containing beef, eggs, and tuna were found to have the highest amount of biotin. Furthermore, results from this study also revealed that lactating persons consuming dietary biotin above the AI experienced a reduction in both plasma and breast-milk biotin concentrations. Moreover, the excretion of urinary bisnorbiotin, a nonfunctional, inactive catabolite from biotin degradation, was higher in lactating individuals than that in pregnant and nonpregnant/nonlactating persons indicating that lactation may be a physiological state that increases biotin catabolism and lactating persons consuming a diet low in biotin may accelerate biotin loss. The dietary biotin requirement during lactation may need to be increased to meet the metabolic demands of lactation and prevent biotin catabolism. For further information, see [5,10,11].

## Author contributions

C.A.P.: writing (original draft preparation), review, edit; T.A.B.: writing, review, edit; all authors have read and agreed to the published version of the manuscript.

## Conflicts of interest

The authors report no conflicts of interest.

## Funding

The authors reported no funding received for this study.

## References

[1] Zempleni J., Kuroishi T. (2012). Biotin. Adv. Nutr..

[2] NIH Office of Dietary Supplements, Biotin: fact sheet for health professionals. https://ods.od.nih.gov/factsheets/Biotin-HealthProfessional/#h7.

[3] Perry C.A., West A.A., Gayle A., Lucas L.K., Yan J., Jiang X. (2014). Pregnancy and lactation alter biomarkers of biotin metabolism in women consuming a controlled diet. J. Nutr..

[4] Perry C.A., Caudill M.A. (2012). Biotin: critical for fetal growth and development yet often overlooked. Nutr. Today.

[5] Institute of Medicine, Biotin, in: Food and Nutrition Board (2006). Dietary reference intakes essential guide to nutrient requirements.

[6] Zempleni J., Liu D., Camara D.T., Cordonier E.L. (2014). Novel roles of holocarboxylase synthetase in gene regulation and intermediary metabolism. Nutr. Rev..

[7] Li Y., Malkaram S.A., Zhou J., Zempleni J. (2014). Lysine biotinylation and methionine oxidation in the heat shock protein HSP60 synergize in the elimination of reactive oxygen species in human cell cultures. J. Nutr. Biochem..

[8] Tourbah A., Lebrun-Frenay C., Edan G., Clanet M., Papeix C., Vukusic S. (2016). MD1003 (high-dose biotin) for the treatment of progressive multiple sclerosis: a randomised, double-blind, placebo-controlled study. Mult. Scler..

[9] Cree B.A.C., Cutter G., Wolinsky J.S., Freedman M.S., Comi G., Giovannoni G. (2020). SPI2 investigative teams, Safety and efficacy of MD1003 (high-dose biotin) in patients with progressive multiple sclerosis (SPI2): a randomised, double-blind, placebo-controlled, phase 3 trial. Lancet Neurol.

[10] Mock D.M. (2017). Biotin: from nutrition to therapeutics. J. Nutr..

[11] Mock D.M., Matthews B.A., Stipanuk M.H., Caudill M.A. (2019). Biotin and pantothenic acid. Biochemical, physiological, and molecular aspects of human nutrition.

